# Miniscrew placement on mandibular buccal shelf and infra-zygomatic crest area: A finite element analysis

**DOI:** 10.34172/japid.025.3687

**Published:** 2025-08-31

**Authors:** Setareh Khosravi, Hosein Jahanshad, Mahsa Moafi, Mohamadreza Shahmohamadi, Mojgan Kachoei

**Affiliations:** ^1^Orthodontics Department, School of Dentistry, Alborz University of Medical Sciences, Karaj, Iran; ^2^Student Research Committe, School of Dentistry, Alborz University of Medical Sciences, Karaj, Iran; ^3^Department of Technology and Science, Coimbra Universitate, Coimbra, Portugal; ^4^Department of Orthodontics, Faculty of Dentistry, Tabriz University of Medical Sciences, Tabriz, Iran

**Keywords:** Buccal shelf, Deformation, Finite element method, Infra-zygomatic crest, Mini-implant, Strain, Stress

## Abstract

**Background.:**

This study investigated the optimal placement of mini-implants in the mandibular buccal shelf and infra-zygomatic crest regions using finite element analysis.

**Methods.:**

Three-dimensional finite element method (FEM) models of bone and mini-implants were created. In the mandibular buccal shelf (MBS) region, mini-screws were positioned at three sites: between the first molar roots, between the second molar roots, and distal to the second molar, tested at two depths (4 mm and 8 mm) and two angles to the occlusal plane (60° and 90°). In the infra-zygomatioc crest (IZC) region, mini-screws were placed between the first and second maxillary molars and adjacent to the mesiobuccal root of the second molar, at depths of 7 mm and 11 mm, and angles of 40° and 75° relative to the occlusal surface. The force of 200 g was applied as immediate loading and in a vertical direction to the center of the miniscrew.

**Results.:**

In the MBS region, the distal second molar site at 8 mm depth and 60° angle exhibited the lowest von Mises stress, while the lowest strain occurred between the first molar roots at the same depth and angle. In the IZC region, the best biomechanical response was found at 7-mm depth between the first and second molars at a 75° angle, with the highest stress occurring near the mesiobuccal root of the second molar at 11-mm depth and 40° angle.

**Conclusion.:**

These findings suggest that for optimal biomechanical performance, MBS mini-screws should be placed distal to the second molar at 4–8-mm depth and 60° angle, and IZC mini-screws between the first and second molars at 7-mm depth and 75° angle.

## Introduction

 Orthodontic treatment often requires additional anchorage to achieve desired tooth movements without relying heavily on patient compliance. Traditional anchorage devices, while effective, have limitations in terms of stability and patient cooperation. The introduction of the miniscrew abutment in 1997 by Kanomi^1^ was a significant improvement over traditional anchorage devices. Today, mini-screws have become widely accepted and are a reliable way to provide temporary additional support during orthodontic treatment,^2^ and are called “temporary anchorage devices” (TADs).^3^ Skeletal anchorage is used to extend the range of tooth movement and requires minimal patient cooperation.^4^ In the evolutionary path of TADs application in orthodontics, there has recently been a tendency to place extra-radicular miniscrews.^5^ Extra-radicular mini-screws with the concept of absolute anchorage have revolutionized the field of orthodontics in the last decade. These anchorage types have even enabled clinicians to convert surgical treatment plans into nonsurgical alternatives without compromising outcomes.^6,7^ Extra-alveolar mini-screws offer several advantages, including a higher probability of success and greater stability. They do not require relocation during treatment, reduce the risk of root damage, allow insertion into areas with more cortical bone, and reduce the number of mini-implants needed to address complex cases.^8^

 The buccal shelf of the mandibular bone and infra-zygomatic crest region are suitable choices for extra-alveolar support in the lower and upper jaws, which greatly expand the range of mechanotherapy.^9,10^ These locations are most helpful in correcting class III and II malocclusions.^5,7,11^ In terms of anatomy, the anatomical borders of the buccal shelf include the alveolar ridge in the medial, the retromolar pad in the distal, the buccal frenum in the mesial, and the external oblique ridge on the lateral side.^12^ The infra-zygomatic crest is the prominence of the zygomatic process that merges with the buccal surface of the maxillary bone. The wide range of recommended areas may be due to local anatomical variations and a lack of studies. Choosing the optimal place to insert the miniscrew in the buccal shelf of the mandible and infra-zygomatic crest region is a significant challenge.

 Engineering has not only advanced in medicine but also profoundly influenced dentistry, especially orthodontics. The finite element method (FEM) is an engineering technique used to calculate stress and strain of complex structures and is widely used in orthodontic research.^13^ FEM helps create a virtual clinical scenario that can be further applied in clinical practice to assess the reliability of a particular procedure.^14^

 The placement of mini-screws includes different modes depending on their location relative to the teeth, the angle relative to the occlusal plane, and the distance from the alveolar crest. Understanding how force is transmitted to mini-screws and cortical and cancellous bone, as well as von Mises stress and micro-strain values, will be valuable for dentists. Most of the studies undertaken to evaluate the buccal shelf area for miniscrew placement are CBCT studies.^11,15^ They have investigated CBCT radiographs without considering von Mises stress and strain values. The studies conducted in the field of finite element analysis on miniscrew placed in the buccal shelf area and infra-zygomatic crest region are few, and most of them have used a bone block for FEM analysis without considering the anatomy of the human jaw.^16^

 Therefore, the purpose of this study was to determine the appropriate position of orthodontic miniscrew placement by finite element analysis method in the buccal shelf area and infra-zygomatic crest with accurate modeling of this area, accurate miniscrew modeling, and evaluation of von Mises stress and micro-strain values.

## Methods

**Figure 1:**
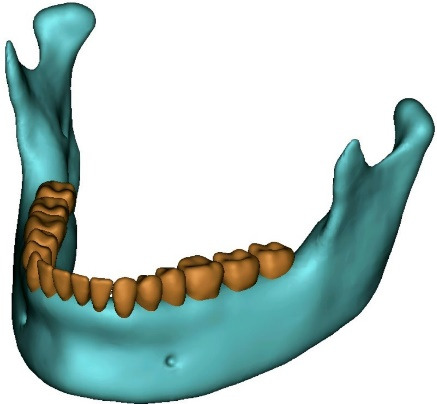
Three-dimensional model of the mandible reconstructed from cone-beam computed tomography (CBCT). This model serves as the basis for subsequent finite element analysis and simulations—model rendered in Mimics 2017

**Figure 2:**
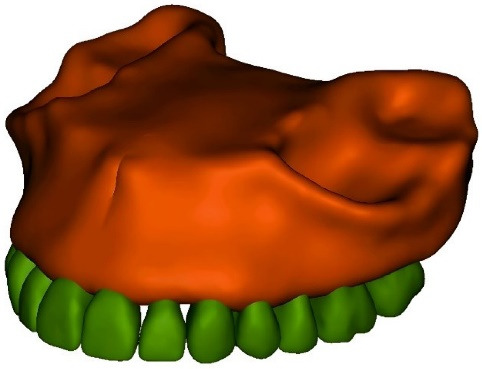
Three-dimensional model of the maxilla reconstructed from cone-beam computed tomography (CBCT). This model serves as the basis for subsequent finite element analysis and simulations—model rendered in Mimics 2017

**Figure 3:**
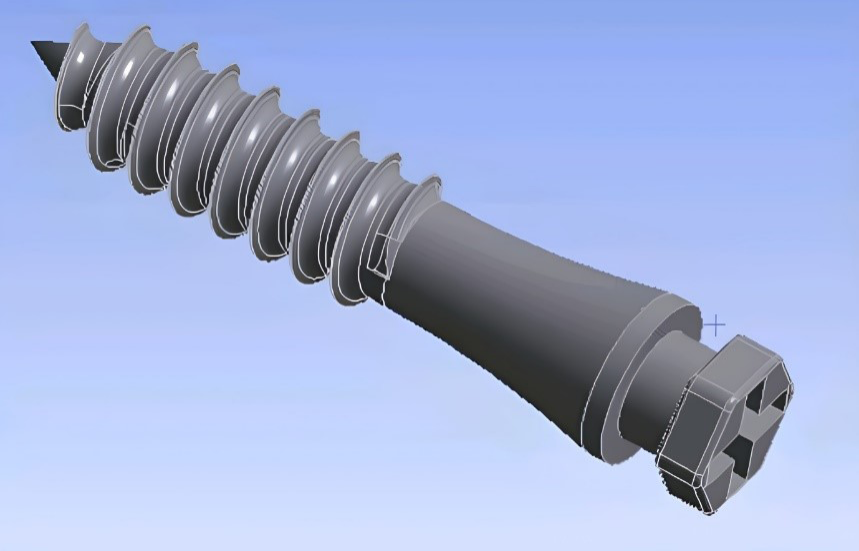
Three-dimensional model of the orthodontic miniscrew (12 × 2 mm JS screw DualTop, Jeil Medical Corporation, Seoul, Korea)—model rendered in Solidworks 2018

**Figure 4:**
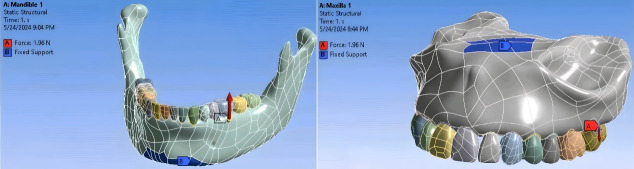
Three-dimensional models of the maxilla and mandible with applied boundary conditions and defined segmentation. The anatomical structures were segmented and meshed for finite element analysis, and boundary constraints were assigned to simulate realistic mechanical conditions—model rendered in Ansys 2018

 Since hard tissues in CT images offer higher contrast compared to soft tissues, these images are more suitable for bone modeling.^17^ For this study, CT scan radiographs were imported into Mimics 20 software (Materialize; Leuven, Belgium), with a slice interval of 1 mm (Figures 1 and 2). The parts were then exported from Mimics 20 in STL format, and subsequently converted to STP format using 3-Matic software (Materialize; Leuven, Belgium). The screw (Figure 3), measuring 12 × 2 mm (JS screw DualTop, Jeil Medical Corporation, Seoul, Korea), was designed using SolidWorks software (version 2018, Dassault Systèmes, Paris, France). Once all geometries were converted to STP format, they were imported into Ansys Workbench 2018 software (ANSYS Inc.; USA) for analysis. The next step in the finite element modeling process involved dividing the model into elements and nodes, a process known as meshing, after which the boundary conditions were defined^18^ (Figure 4). All the materials used in the study were assumed to be linearly elastic, homogeneous, and isotropic.^19^ The material properties for both the model and miniscrew were assigned based on data from relevant literature sources^20^ (Table 1).

###  Miniscrew placement on buccal shelf of the mandible

**Figure 5:**
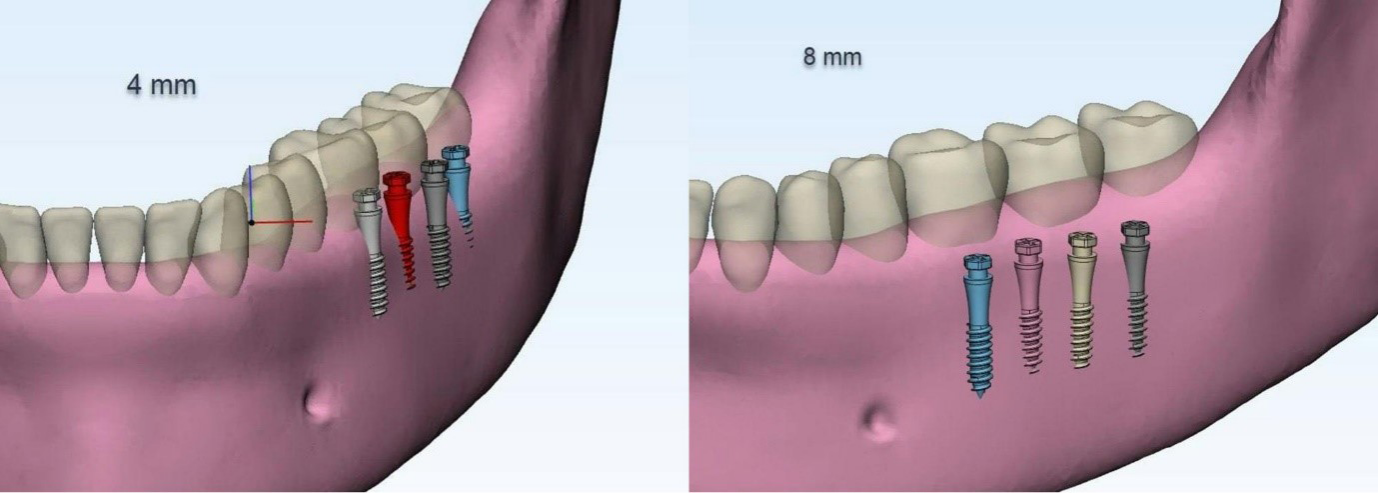
The mini-screws are positioned at a 90° angle and placed at 4 mm and 8 mm from the alveolar crest in various locations, except at the distal aspect of the second molar, where they are only placed at 4 mm from the crest—model rendered in Ansys 2018

 Mini-screws were virtually placed in three locations on the buccal shelf of the mandible: between the mesial and distal roots of the first mandibular molar,^15^ between the mesial and distal roots of the second mandibular molar,^21^ and on the distal side of the second mandibular molar.^21^ In each of these locations, mini-screws were placed at two distances of 4 and 8 mm from the alveolar crest,^9^ at a 60° angle to the occlusal plane.^15^ However, in the distal second molar area, due to anatomical changes and the amount of bone available, it was possible to place the miniscrew at a 90° angle^15^ and a distance of 4 mm. As a result, seven different positions were formed for the miniscrew in the buccal area of the mandibular shelf. Due to the lack of sufficient bone, the mini-screws were exposed at a 90° angle and at a distance of 4 and 8 mm from the alveolar crest in different locations except for the distal second molar at a distance of 4 mm from the crest (Figure 5).

 The seven different miniscrew positions are as follows:

1. M6_60_4: Between the mesial and distal roots of the first mandibular molar, at a 60° angle to the occlusal plane and a distance of 4 mm from the alveolar crest
2. M6_60_8: Between the mesial and distal roots of the first mandibular molar, at a 60° angle to the occlusal plane and a distance of 8 mm from the alveolar crest
3. M7_60_4: Between the mesial and distal roots of the second mandibular molar, at a 60° angle to the occlusal plane and a distance of 4 mm from the alveolar crest
4. M7_60_8: Between the mesial and distal roots of the second mandibular molar, at a 60° angle to the occlusal plane and a distance of 8 mm from the alveolar crest
5. DM7_60_4: On the distal side of the second mandibular molar, at a 60° angle to the occlusal plane and a distance of 4 mm from the alveolar crest
6. DM7_60_8: On the distal side of the second mandibular molar, at a 60° angle to the occlusal plane and a distance of 8 mm from the alveolar crest
7. DM7_90_4: On the distal side of the second mandibular molar, at a 90° angle to the occlusal plane and a distance of 4 mm from the alveolar crest

###  Miniscrew placement on the infra-zygomatic crest

 Mini-screws were virtually placed in two locations on the infra-zygomatic crest: between the mesiobuccal root of the second maxillary molar and the distobuccal root of the first maxillary molar, and adjacent to the mesiobuccal root of the second maxillary molar. In each of these locations, the miniscrew was placed at two distances: 7 mm and 11 mm from the alveolar crest, and at two angles: 40° and 75° relative to the occlusal surface.^22,23^ However, when placed at a distance of 7 mm from the alveolar crest and at both angles (40° and 75°) near the mesiobuccal root of the second maxillary molar, the miniscrew collided with the tooth roots. Therefore, a total of six positions were considered for miniscrew placement in the infra-zygomatic crest area. Therefore, a total of six positions were considered for miniscrew placement in the infra-zygomatic crest area :

1. Between the mesiobuccal root of the second maxillary molar and the distobuccal root of the first maxillary molar, 7 mm distance from the alveolar crest, 40º relative to the occlusal plane
2. Between the mesiobuccal root of the second maxillary molar and the distobuccal root of the first maxillary molar, 7 mm distance from the alveolar crest, 75º relative to the occlusal plane
3. Between the mesiobuccal root of the second maxillary molar and the distobuccal root of the first maxillary molar, 11 mm distance from the alveolar crest, 40º relative to the occlusal plane
4. Between the mesiobuccal root of the second maxillary molar and the distobuccal root of the first maxillary molar, 11 mm distance from the alveolar crest, 75º relative to the occlusal plane
5. The mesiobuccal root of the second maxillary molar, 11 mm distance from the alveolar crest, 40º relative to the occlusal plane
6. The mesiobuccal root of the second maxillary molar, 11 mm distance from the alveolar crest, 40º relative to the occlusal plane

###  Force application and immediate loading

 To simulate the reaction force of the spring, based on previous studies,^5,12^ a 200-g force (equivalent to 1.96 Newtons) was applied as immediate loading and in a vertical direction to the center of the miniscrew. After creating the model and applying boundary conditions and external loads, the system solved the equations and extracted von Mises stress, strain, and deformation data.

## Results

Table 2 summarizes the results of placing the miniscrew on the buccal shelf, including von Mises stress and strain on bone around the miniscrew.

**Figure 6:**
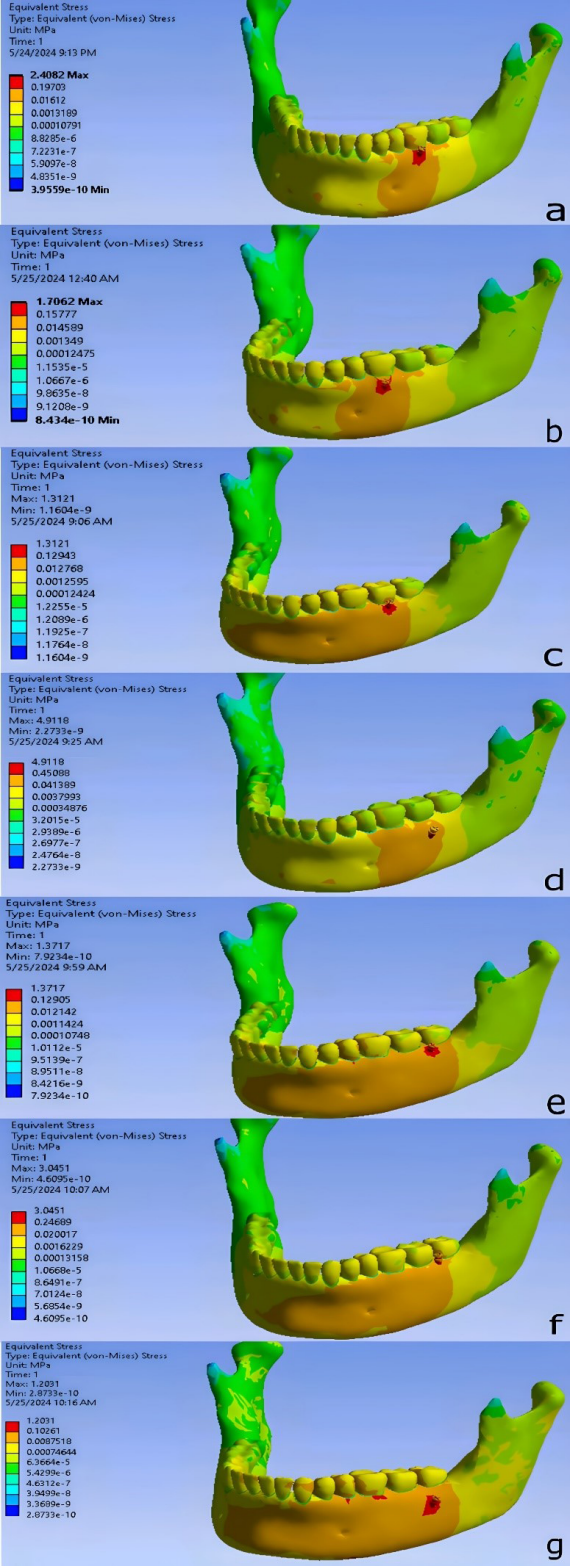
Stress distribution pattern in different regions of the mandible. (a) Between the mesial and distal roots of the first mandibular molar, at a 60° angle to the occlusal plane and a distance of 4 mm from the alveolar crest. (b) Between the mesial and distal roots of the first mandibular molar, at a 60° angle to the occlusal plane and a distance of 8 mm from the alveolar crest. (c) Between the mesial and distal roots of the second mandibular molar, at a 60° angle to the occlusal plane and a distance of 4 mm from the alveolar crest. (d) Between the mesial and distal roots of the second mandibular molar, at a 60° angle to the occlusal plane and a distance of 8 mm from the alveolar crest. (e) On the distal side of the second mandibular molar, at a 60° angle to the occlusal plane and a distance of 4 mm from the alveolar crest. (f) On the distal side of the second mandibular molar, at a 60° angle to the occlusal plane and a distance of 8 mm from the alveolar crest. (g) On the distal side of the second mandibular molar, at a 90° angle to the occlusal plane and a distance of 4 mm from the alveolar crest—models rendered in Ansys 2018

 The lowest stress level in bone among the various miniscrew placement positions was observed in DM7_60_8, while the highest stress level was recorded in M7_60_8. The von Mises stress contours for each placement position are illustrated in Figures 6a-6g.

**Figure 7:**
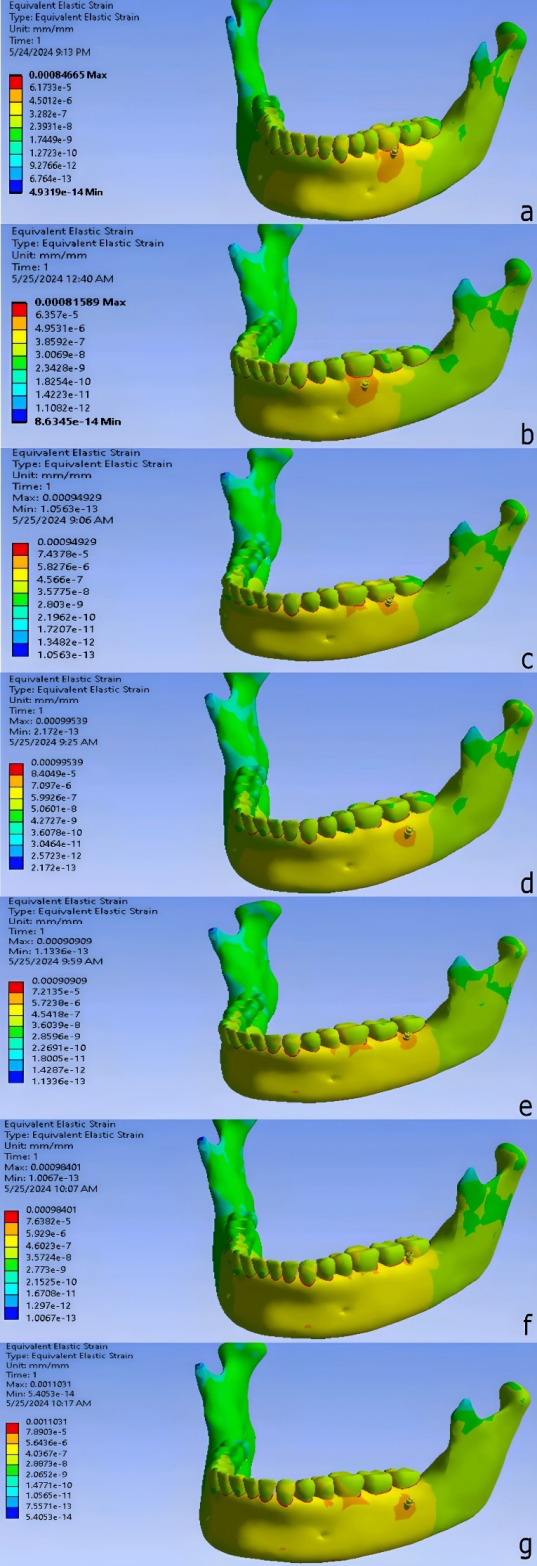
Strain distribution pattern in different regions of the mandible. (a) Between the mesial and distal roots of the first mandibular molar, at a 60° angle to the occlusal plane and a distance of 4 mm from the alveolar crest. (b) Between the mesial and distal roots of the first mandibular molar, at a 60° angle to the occlusal plane and a distance of 8 mm from the alveolar crest. (c) Between the mesial and distal roots of the second mandibular molar, at a 60° angle to the occlusal plane and a distance of 4 mm from the alveolar crest. (d) Between the mesial and distal roots of the second mandibular molar, at a 60° angle to the occlusal plane and a distance of 8 mm from the alveolar crest. (e) On the distal side of the second mandibular molar, at a 60° angle to the occlusal plane and a distance of 4 mm from the alveolar crest. (f) On the distal side of the second mandibular molar, at a 60° angle to the occlusal plane and a distance of 8 mm from the alveolar crest. (g) On the distal side of the second mandibular molar, at a 90° angle to the occlusal plane and a distance of 4 mm from the alveolar crest—models rendered in Ansys 2018

 The lowest strain was observed in M6_60_8, while the highest strain was found in DM7_60_8 across the different miniscrew placements (Figures 7a-7g).

 In M6_60_4, the highest von Mises stress observed around the miniscrew was 2.4082 MPa, and the highest strain was 0.00084665 mm/mm.

 In M6_60_8, the highest von Mises stress was 1.7062 MPa, and the highest strain was 0.00081589 mm/mm.

 In M7_60_4, the highest von Mises stress recorded around the miniscrew was 1.3121 MPa, and the highest strain was 0.00094929 mm/mm.

 In M7_60_8, the highest von Mises stress was 4.9118 MPa, and the highest strain was 0.00099539 mm/mm.

 In DM7_60_4, the highest von Mises stress observed was 1.3717 MPa, and the highest strain was 0.00090909 mm/mm.

 In DM7_60_8, the highest von Mises stress was 1.2031 MPa, and the highest strain was 0.0011031 mm/mm.

 In DM7_90_4, the highest von Mises stress observed around the miniscrew was 3.0451 MPa, and the highest strain was 0.00098401 mm/mm.

**Figure 8:**
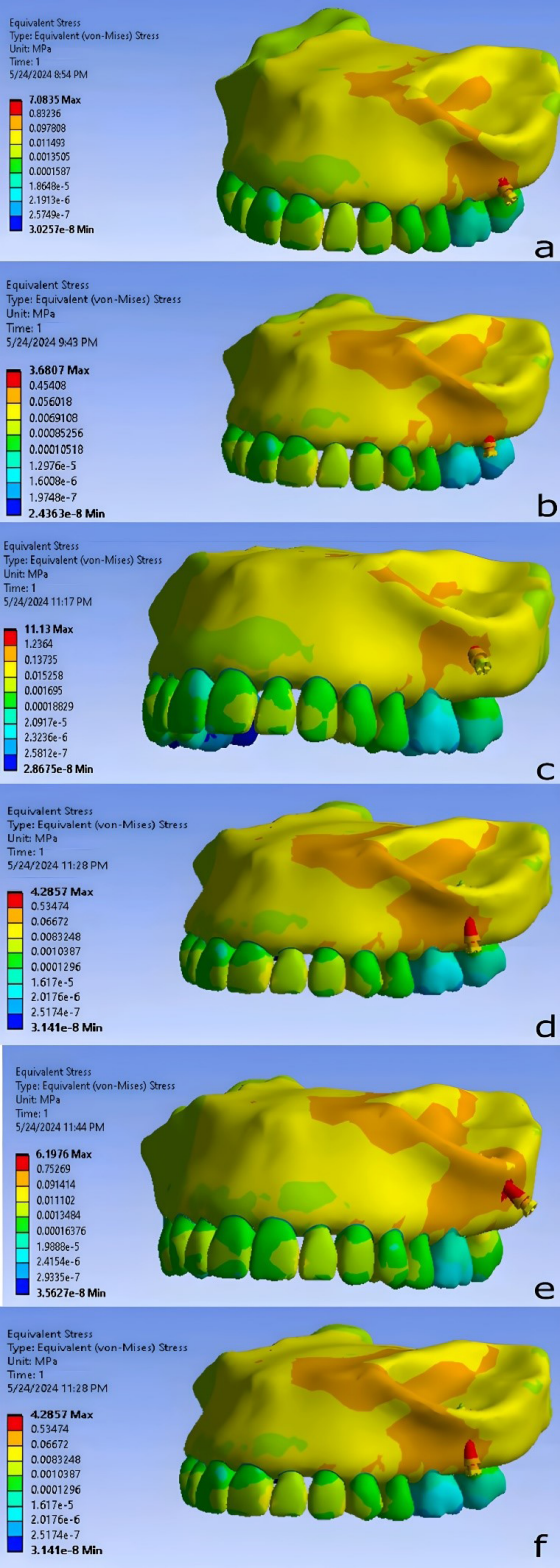
Stress distribution pattern in different regions of the infra-zygomatic crest. (a) Between the mesiobuccal root of the second maxillary molar and the distobuccal root of the first maxillary molar, 7-mm distance from the alveolar crest, 40º relative to the occlusal plane. (b) Between the mesiobuccal root of the second maxillary molar and the distobuccal root of the first maxillary molar, 7-mm distance from the alveolar crest, 75º relative to the occlusal plane. (c) Between the mesiobuccal root of the second maxillary molar and the distobuccal root of the first maxillary molar, 11-mm distance from the alveolar crest, 40º relative to the occlusal plane. (d) Between the mesiobuccal root of the second maxillary molar and the distobuccal root of the first maxillary molar, 11-mm distance from the alveolar crest, 75º relative to the occlusal plane. (e) The mesiobuccal root of the second maxillary molar, 11-mm distance from the alveolar crest, 40º relative to the occlusal plane. (f) The mesiobuccal root of the second maxillary molar, 11-mm distance from the alveolar crest, 40º relative to the occlusal plane—models rendered Ansys 2018

**Figure 9:**
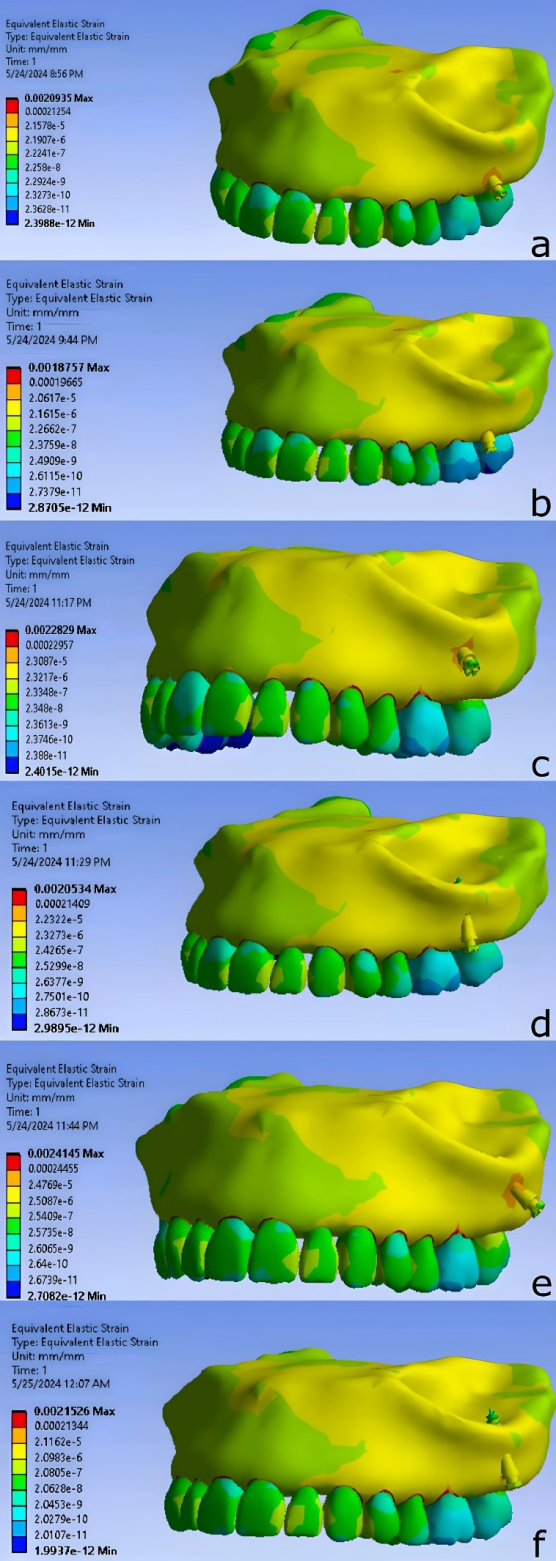
Strain distribution pattern in different regions of the infra-zygomatic crest. (a) Between the mesiobuccal root of the second maxillary molar and the distobuccal root of the first maxillary molar, 7-mm distance from the alveolar crest, 40º relative to the occlusal plane. (b) Between the mesiobuccal root of the second maxillary molar and the distobuccal root of the first maxillary molar, 7-mm distance from the alveolar crest, 75º relative to the occlusal plane. (c) Between the mesiobuccal root of the second maxillary molar and the distobuccal root of the first maxillary molar, 11-mm distance from the alveolar crest, 40º relative to the occlusal plane. (d) Between the mesiobuccal root of the second maxillary molar and the distobuccal root of the first maxillary molar, 11-mm distance from the alveolar crest, 75º relative to the occlusal plane. (e) The mesiobuccal root of the second maxillary molar, 11-mm distance from the alveolar crest, 40º relative to the occlusal plane. (f) The mesiobuccal root of the second maxillary molar, 11-mm distance from the alveolar crest, 40º relative to the occlusal plane—models rendered Ansys 2018

**Figure 10:**
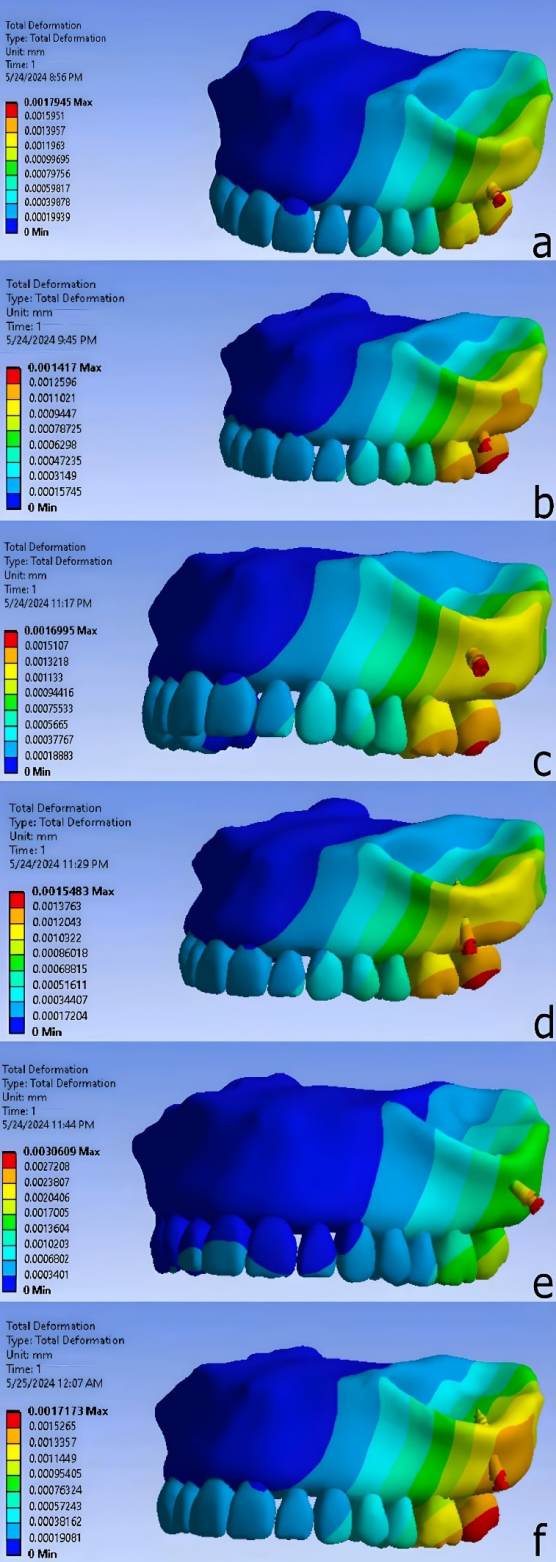
Deformation distribution pattern in different regions of the infra-zygomatic crest. (a) Between the mesiobuccal root of the second maxillary molar and the distobuccal root of the first maxillary molar, 7-mm distance from the alveolar crest, 40º relative to the occlusal plane. (b) Between the mesiobuccal root of the second maxillary molar and the distobuccal root of the first maxillary molar, 7-mm distance from the alveolar crest, 75º relative to the occlusal plane. (c) Between the mesiobuccal root of the second maxillary molar and the distobuccal root of the first maxillary molar, 11-mm distance from the alveolar crest, 40º relative to the occlusal plane. (d) Between the mesiobuccal root of the second maxillary molar and the distobuccal root of the first maxillary molar, 11-mm distance from the alveolar crest, 75º relative to the occlusal plane. (e) The mesiobuccal root of the second maxillary molar, 11-mm distance from the alveolar crest, 40º relative to the occlusal plane. (f) The mesiobuccal root of the second maxillary molar, 11-mm distance from the alveolar crest, 40º relative to the occlusal plane—models rendered Ansys 2018

 The results of miniscrew placement in the infra-zygomatic crest, including von Mises stress, strain, and deformation values, are presented as contours in Figures 8-10 and summarized numerically in Table 3.

**Table 1:** Mechanical properties of the materials/structures used in the current study

**Component**	**Young’s module (GPa)**	**Poisson’s ratio**
Micro implant	110	0.36
Cortical bone	14.7	0.3
Cancellous bone (D2)	5.5	0.3

**Table 2:** Von Mises stress and strain in the mandibular buccal shelf region for each miniscrew position

**Miniscrew position**	**Max von Mises stress (MPa)**	**Max strain (mm/mm)**
M6_60_4^1^	2.4082	0.00084665
M6_60_8^2^	1.7062	0.00081589
M7_60_4^3^	1.3121	0.00094929
M7_60_8^4^	4.9118	0.00099539
DM7_60_4^5^	1.3717	0.00090909
DM7_60_8^6^	1.2031	0.0011031
DM7_90_4^7^	3.0451	0.00098401

^1 ^Between the mesial and distal roots of the first mandibular molar at an angle of 60º to the occlusal plane and at a distance of 4 mm from the alveolar crest. ^2 ^Between the mesial and distal roots of the first molar of the mandible at an angle of 60º to the occlusal plane and at a distance of 8 mm from the alveolar crest. ^3 ^Between the mesial and distal roots of the second molar of the mandible at an angle of 60º to the occlusal plane and at a distance of 4 mm from the alveolar crest. ^4 ^Between the mesial and distal roots of the second molar of the mandible at an angle of 60º to the occlusal plane and at a distance of 8 mm from the alveolar crest. ^5 ^On the distal side of the second molar of the mandible at an angle of 60º to the occlusal plane and at a distance of 4 mm from the alveolar crest. ^6 ^On the distal side of the second molar of the mandible at an angle of 60º to the occlusal plane and at a distance of 8 mm from the alveolar crest. ^7 ^On the distal side of the second molar of the mandible at a 90º angle to the occlusal plane and at a distance of 4 mm from the alveolar crest.

**Table 3:** von Mises stress, strain, and deformation in the mentioned position in the infra-zygomatic crest

**Miniscrew position**	**Max von Mises stress (MPa)**	**Max strain (mm/mm)**	**Max deformation (mm)**
U6_U7 / 7 mm / 40º ^1^	7.0835	0.0020935	0.0017945
U6_U7 / 7 mm / 75º ^2^	3.6807	0.0018757	0.001417
U6_U7 / 11 mm / 40º ^3^	11.13	0.0022829	0.0016995
U6_U7 / 11 mm / 75º^4^	4.2857	0.0020534	0.0015483
U7 / 11 mm / 40º ^5^	6.1976	0.0024145	0.0030609
U7 / 11 mm / 75º^6^	4.0014	0.0021526	0.001717

^1 ^Between the mesiobuccal root of the second maxillary molar and the distobuccal root of the first maxillary molar, 7-mm distance from the alveolar crest, 40º relative to the occlusal plane. ^2 ^Between the mesiobuccal root of the second maxillary molar and the distobuccal root of the first maxillary molar, 7-mm distance from the alveolar crest, 75º relative to the occlusal plane. ^3 ^Between the mesiobuccal root of the second maxillary molar and the distobuccal root of the first maxillary molar, 11-mm distance from the alveolar crest, 40º relative to the occlusal plane. ^4 ^Between the mesiobuccal root of the second maxillary molar and the distobuccal root of the first maxillary molar, 11-mm distance from alveolar crest, 75º relative to occlusal plane. ^5 ^The mesiobuccal root of the second maxillary molar, 11-mm distance from the alveolar crest, 40º relative to the occlusal plane. ^6 ^The mesiobuccal root of the second maxillary molar, 11-mm distance from alveolar crest, 40º relative to occlusal plane.

 The lowest von Mises stress in bone was observed when the miniscrew was placed between the distobuccal root of the first molar and the mesiobuccal root of the second molar, at a distance of 7 mm from the alveolar crest and an angle of 75°. The second lowest stress level occurred when the miniscrew was placed between the roots of the first and second molars, at a distance of 11 mm from the alveolar crest and an angle of 75°. The highest stress was found when the miniscrew was placed between the distobuccal root of the first molar and the mesiobuccal root of the second molar, at a distance of 11 mm from the alveolar crest and an angle of 40° relative to the occlusal plane.

 The miniscrew placed between the distobuccal root of the first molar and the mesiobuccal root of the second molar, at a distance of 7 mm from the alveolar crest and at an angle of 75° relative to the occlusal plane, resulted in the lowest strain level. Conversely, the highest strain level was observed when the miniscrew was placed at the mesiobuccal root of the second molar, at a distance of 11 mm from the alveolar crest and at an angle of 40° relative to the occlusal plane.

 The lowest deformation level among the various miniscrew placement positions was observed when the miniscrew was placed between the distobuccal root of the first molar and the mesiobuccal root of the second molar, at a distance of 7 mm from the alveolar crest and at an angle of 75° relative to the occlusal plane. The highest deformation level was found when the miniscrew was placed at the mesiobuccal root of the second molar, at a distance of 11 mm from the alveolar crest and at an angle of 40° relative to the occlusal plane.

 The stress distribution was similar in all models, with maximum stress concentrated at the implant neck and adjacent bone. The greatest deformation was observed at the implant head. Strain was primarily concentrated in the interproximal alveolar space, with the second highest levels near the implant neck.

## Discussion

 Finite element analysis (FEA), also known as the FEM, is an engineering technique that works by dividing a structure into a finite number of small elements.^13^ In this study, we evaluated mechanical conditions such as von Mises stress, strain, and deformation, and simulated clinical scenarios using FEA in various miniscrew placement positions in the buccal shelf area of the mandibular bone and infra-zygomatic crest area. We tested different positions and examined variables such as miniscrew location relative to the teeth, the distance from the alveolar crest, and the angle to the occlusal plane.

 Placing a miniscrew at a 90° angle in the buccal shelf area poses significant risks. In most cases, except in the distal second molar area and at a distance of 4 mm from the alveolar crest, there is insufficient bone for successful miniscrew insertion. Exposure of the screw threads from the bone often leads to miniscrew failure and, consequently, orthodontic treatment failure.^24^ Clinicians should take necessary precautions when considering a 90° placement and use template guides for precision.

 In the infra-zygomatic crest, mini-screws interfered with the mesiobuccal root of the second molar in two specific scenarios: at a distance of 7 mm and an angle of 40°, and at a distance of 7 mm and an angle of 75°, both adjacent to the mesiobuccal root. These placements carry a significant clinical risk of root contact, and based on our findings, placing a miniscrew in these areas is not recommended.

 ANSYS software calculations in our study showed that the lowest stress around the MBS miniscrew, when applying an immediate force of 200 g,^5,12^ was observed in the distal second molar area, at a distance of 8 mm from the alveolar crest and an angle of 60° to the occlusal plane. The distal second molar area is located further back compared to the other two areas (between the mesial and distal roots of the first molar and between the mesial and distal roots of the second molar). As we move posteriorly and basally in the buccal shelf of the mandible, both cortical bone thickness and total bone thickness increase, leading to improved secondary stability and a decrease in stress on the surrounding area.^15,21^

 These findings suggest that the posterior and basal regions of the mandible provide a more stable environment for miniscrew placement. The increased bone thickness and cortical support in the distal second molar area contribute to better stress distribution, reducing the risk of failure.

 In terms of location relative to the teeth, these findings are consistent with results of CBCT-based studies that have identified the distal region of the second molar as the optimal placement site for mini-screws in the buccal shelf, considering variables such as age, growth pattern, and gender.^25^ A similar CBCT-based evaluation assessing multiple locations, insertion angles, and distances from the CEJ also indicated a progressive increase in cortical bone thickness from the first to the second molar.^15^ Likewise, findings from CBCT analyses confirmed that the buccal area of the mandibular bone shelf is generally thicker in the distal second molar region,^21^ in agreement with the present study.

 Based on CBCT evaluations,^26^ the best location for miniscrew placement in the mandibular buccal shelf is at the distal root of the second molar. Kolge et al^27^ also used CBCT imaging to conclude that the most suitable area for placing mini-screws is the distal second molar region, due to adequate root clearance, minimal cheek tissue irritation, and greater bone width. Finally, CBCT-based studies by Elshebiny et al^9^ and Nucera et al^28^ confirmed that the distal second molar region offers the most favorable anatomical conditions for miniscrew placement.

 Our results align with these anatomical observations, indicating that the distal second molar region offers more favorable conditions for miniscrew placement due to its thicker cortical bone and more stable structure.

 In terms of distance from the alveolar crest, our study found that the lowest stress was around the miniscrew placed in the distal second molar region, at a distance of 8 mm from the alveolar crest and an angle of 60° to the occlusal plane. An 8-mm distance is more apical compared to a 4-mm distance, and several studies support the notion that moving towards the apical side of the buccal shelf leads to a greater presence of cortical bone.^15,21^

 A CBCT-based study also concluded that the thickness of the mandibular bone increases posteriorly and basally, which aligns with our findings.^21^ Similarly, Patla et al,^29^ through CBCT analyses, showed that the maximum bone thickness is found in the distal region of the second molar at a distance of 8 mm from the CEJ. This finding also supports Elshebiny et al’s^9^ CBCT findings regarding the ideal location (distal to the second molar) and the 8-mm distance from the CEJ.

 These findings indicate that placing the miniscrew further from the alveolar crest, in the apical direction, enhances the bone thickness, which contributes to a more stable anchorage and reduced stress.

 In terms of angle to the occlusal plane, the lowest stress was observed around the miniscrew placed in the distal second molar region, at a distance of 8 mm from the alveolar crest and at an angle of 60°. This finding is consistent with the FEA, which reported that a 60° angle resulted in the lowest von Mises stress.^30^ Additionally, another FEA study,^31^ demonstrated that a 30° angle relative to the bone surface produces less stress and strain in the cortical bone, which is consistent with our findings, as 30° from the bone surface is approximately the same as 60° relative to the occlusal plane. A CBCT-based study^11^ found that a 30° angle from the tooth’s long axis is most appropriate, which corresponds to 60° from the occlusal plane.

 In contrast, a CBCT-based study^32^ suggested that the best placement angle for the miniscrew in the buccal shelf is parallel to the long axis of the tooth, as this results in greater engagement with the cortical bone. However, this study was conducted using CBCT and did not evaluate von Mises stress values.

 These results suggest that a 60° angle to the occlusal plane offers the optimal balance of stress and strain, supporting the mechanical stability of the miniscrew.

 In general, most studies evaluating the buccal shelf area for the placement of extra-alveolar mini-screws have focused on assessing bone thickness. However, the number of FEM studies in this area is very limited. Investigations of FEM and von Mises values, such as the study by Cozzani et al,^16^ which used a bone block without considering anatomical conditions, or the study by Arash Poorsattar Bejeh Mir et al,^31^ which measured von Mises values for skeletal anchorage from a bone model derived from the palatal region, do not account for the structural and anatomical differences that influence stress and strain values such as variations in cortical and cancellous bone in areas like the buccal shelf. Additionally, most finite element analysis studies in dentistry do not evaluate strain values. In our study, we observed that the lowest strain occurred in the “M6_60_8” position, while the highest strain was found in the “M7_60_8” position.

 von Mises stress is a scalar measure that combines the principal stress components along the x, y, and z axes into an equivalent uniaxial stress, and is widely used in ductile material analysis to predict yielding under complex loading conditions. In contrast, strain—especially equivalent strain—quantifies the actual deformation (tensile, compressive, bending, or shear) a material experiences, and is directly influenced by the magnitude, type, and orientation of applied stresses. Unlike von Mises stress, equivalent strain depends on how stresses are applied at different angles and cannot be obtained by simply summing stress components. As such, von Mises stress and equivalent strain are inherently different: while von Mises predicts yielding, it does not fully describe material deformation, which varies with loading mode and orientation. Therefore, these two metrics are not necessarily aligned and should be interpreted separately in multiaxial analyses.^33^

 In the “DM7_60_4” position, both stress and strain values were lower compared to other positions, suggesting that this region could also be suitable for miniscrew placement.

 FEM calculations in the ANSYS software environment showed that in infra-zygomatic crest region the lowest levels of stress, strain, and deformation around the miniscrew, when a 200-g immediate force was applied in the vertical axis, were found in the area between the first and second molars, at a distance of 7 mm from the alveolar crest and an angle of 75º (U6_U7/75/7MM). Additionally, in this scenario, the mini-screw did not interfere with the maxillary sinus space. However, slight intrusion of the mini-screw into this space does not jeopardize its prognosis.^21,22^ It is noteworthy that the mini-screw has a small distance from the distobuccal roots of the first molar and the mesiobuccal roots of the second molar, so this situation may not be the same in all individuals. This highlights the necessity of using CBCT images and guide templates for the accurate placement of mini-screws.

 With a slight difference from this scenario, the stress levels were lowest in the mesiobuccal root of the second maxillary molar, 11 mm away from alveolar crest, 40° relative to occlusal plane (U7/75/11MM) and between the mesiobuccal root of the second maxillary molar and the distobuccal root of the first maxillary molar, 11 mm away from the alveolar crest, 75° relative to the occlusal plane (U6_U7/75/11MM), respectively. However, in these two scenarios, a slight intrusion of the mini-screw into the maxillary sinus space was observed, with the intrusion being more significant in the U7/75/11MM condition.

 The stress distribution pattern was similar in all scenarios, with the highest stress observed in the neck area and adjacent to the bone surface. This differs for deformation and strain. Upon examining the deformation pattern, the highest amount was seen in the head region. For the strain distribution pattern, the highest amount was in the empty space between the teeth and the alveolar process, which was not the focus of this study. However, the second highest strain was observed in the bone adjacent to the neck area of the mini-implant.

 The location between the first and second molars, which in our study had the least stress compared to other scenarios, aligns with the study by Liu et al.^22^ They concluded that the best area for mini-screw placement is between teeth #6 and #7, at a distance of 11 mm from the alveolar crest in the infra-zygomatic crest area. The observed lowest stress at this location can be attributed to the anatomical characteristics of the region. Specifically, this area tends to have thicker cortical bone, which allows for more stable mini-screw placement and better distribution of applied forces, minimizing stress concentrations. This anatomical advantage likely leads to lower mechanical stress, reducing the risk of screw failure.

 According to Lima et al,^23^ a distance of 11 mm from the crest between the first and second maxillary molars is safe for all three facial types (convex, normal, and concave).The findings of our study confirm that an 11 mm distance from the alveolar crest provides sufficient bone thickness and stability for mini-screw placement, reducing the risk of stress accumulation.

 A CBCT-based study was conducted to determine the optimal insertion angle and location for mini-screw placement in the infra-zygomatic crest area adjacent to the distobuccal root of the first molar. It concluded that the best location for mini-screw placement is 12‒17 mm above the occlusal plane, at an angle of 65‒70º.^23^ The similarity of our findings, particularly the lower stress levels at a 75° angle, indicates that the angle and placement location play a significant role in minimizing mechanical stresses. The choice of an angle between 65° and 75° helps in better distributing the applied forces and preventing localized stress concentrations.

 Previous studies in this field were limited to tomographic studies using CBCT, and they attempted to determine the optimal location for mini-screw placement in the infra-zygomatic crest area merely by considering bone thickness at different distances. They did not evaluate stress and strain. In contrast, our study’s use of FEA provides a more comprehensive analysis by assessing not only bone thickness but also the mechanical responses (stress and strain), offering a more accurate representation of the conditions during mini-screw placement.

 The available studies in this field using FEA are based solely on a bone block model without considering anatomical details, such as the study conducted by Paul et al^8^ in 2021. However, the bone surface in the infra-zygomatic crest area differs from that of a bone block. Additionally, the anatomical slope of this area varies at different distances from the alveolar crest, which can explain the observed differences. Our study’s inclusion of the actual anatomical variation of the region allows for more realistic simulation results, leading to a more precise understanding of the forces at play.

 Among the results obtained, models 2, 4, and 6 had a 75º angle. Compared to the scenarios with a 40º angle relative to the occlusal plane, they exhibited lower levels of stress, strain, and deformation. The use of a 75° angle helps in optimizing the load distribution along the mini-screw, leading to a reduction in localized stress and deformation. This finding supports the recommendation of using this angle in clinical practice.

 The limitations of this study were that, to compensate for the lack of knowledge about bone tissues and their behavior, both cortical and cancellous bones were considered homogeneous, linear elastic, and isotropic. However, in reality, this is not the case. In addition, in the construction of the geometry of this model, soft tissue simulation was not considered. For further studies, it is suggested to include stress transfer in adjacent structures such as tooth roots.

## Conclusion

 This study indicated that the optimal position for mini-screw placement in the buccal shelf of the mandible is distal to the second molar region, at distances of 4 and 8 mm from the alveolar crest, with a 60° angle to the occlusal plane. The only safe zone for placing the mini-screw at a 90° angle to the occlusal plane is also distal to the second molar region, at a 4-mm distance from the crest.

 In the infra-zygomatic crest area, a 75° angle results in lower levels of stress, strain, and deformation compared to a 40° angle across all regions. The optimal scenario is between the first and second maxillary molars, at a distance of 7 mm from the alveolar crest and a 75° angle. Additionally, placing the mini-screw adjacent to the mesiobuccal root of the second molar at a height of 11 mm is viable and can be considered by specialists.

## Competing Interests

 The authors declare that they have no competing interests.

## Data Availability

 All data are included in this published article.

## Ethical Approval

 This study was approved by the ethics committee under the approval codes of IR.ABZUMS.REC.1402.247 and IR.ABZUMS.REC.1402.248.
